# Comparison of Pediatric Acute Appendicitis Before and During the COVID-19 Pandemic in New York City

**DOI:** 10.5811/westjem.59393

**Published:** 2023-08-22

**Authors:** Priya Mallikarjuna, Saikat Goswami, Sandy Ma, Won Baik-Han, Kelly L. Cervellione, Gagan Gulati, Lily Q. Lew

**Affiliations:** * Flushing Hospital Medical Center, Department of Pediatrics, Flushing, New York; † Medisys Health Network, Department of Clinical Research, Jamaica, New York

## Abstract

**Background:**

Acute appendicitis (AA) is the most common abdominal surgical emergency in children and adolescents. In the year immediately following the declaration of the coronavirus disease 2019 (COVID-19) pandemic by the World Health Organization (WHO), there was a precipitous decline in emergency department (ED) visits especially for surgical conditions and infectious diseases. Fear of exposure to severe acute respiratory coronavirus 2 infection resulted in delay in presentation and time to surgery, and a shift toward more conservative management.

**Objective:**

Our goal was to compare the incidence and severity of AA before and during the COVID-19 pandemic.

**Methods:**

Patients aged 2–18 years admitted with the diagnosis of AA to Flushing Hospital Medical Center or Jamaica Hospital Medical Center in Queens, New York, were selected for chart review. Data extracted from electronic health records included demographics, clinical findings, imaging studies, and operative and pathological findings. We calculated the Alvarado score (AS) for incidence and the American Association for the Surgery of Trauma (AAST) grade for severity. We compared patients admitted between March 1, 2018–February 29, 2020 (pre-pandemic) to patients admitted between March 1, 2020–February 28, 2021 (pandemic). We then compared pre-pandemic and pandemic groups to determine differences in pediatric AA incidence and severity.

**Results:**

Of 239 patients diagnosed with AA, 184 (77%) were in the pre-pandemic group and 55 (23%) in the pandemic group. Incidence (number per year) of AA declined by 40%. The pandemic group had significantly greater overall AS of ≥7, indicating increased likelihood to require surgery, (*P* = 0.04) and higher AAST grade demonstrating increased severity (***P*** **= 0.02**).

**Conclusion:**

There was a decline in the number of AA cases seen in our pediatric EDs and admitted during the first year of the pandemic. Clinicians need to be aware of increased severity of AA at time of presentation during public health emergencies such as a pandemic, possibly due to modified patient behavior.

## INTRODUCTION

In March 2020 the first case of coronavirus disease 2019 (COVID-19) was identified in New York City (NYC), and the borough of Queens became the epicenter within days. Following the World Health Organization (WHO) declaration of a global pandemic, practices to reduce the spread of severe acute respiratory syndrome coronavirus 2 (SARS-CoV-2) included closing all nonessential businesses and schools, use of personal protective equipment, social distancing, and density reduction.^1^ Routine pediatric and emergency department (ED) visits during the pandemic declined precipitously.^2^ Acute appendicitis (AA), the most common abdominal surgical emergency in pediatrics, was impacted. There is a paucity of data on pediatric ED visits in urban teaching community hospitals and on whether the trends affected presentation or progression of AA in this type of setting. We compared the Alvarado score (AS) for incidence and the American Association for the Surgery of Trauma (AAST) grade for severity in patients with AA before and during the first year of the COVID-19 pandemic.

## METHODS

The institutional review boards of Flushing Hospital Medical Center (FHMC) and Jamaica Hospital Medical Center (JHMC) approved the study. We analyzed charts of patients meeting inclusion criteria who were seen and admitted with AA to either FHMC or JHMC. Data extracted from electronic health records included demographics, clinical presentation, imaging studies, and operative and pathological criteria to determine the AS and AAST grade. Demographics included age, gender, and ethnicity. The AS uses a 10-point clinical scoring system to identify AA based on criteria such as migration of pain, anorexia, nausea, tenderness in right lower quadrant, rebound, fever, leukocytosis and left shift of leukocytosis. Right lower quadrant pain and leukocytosis are assigned two points and the remaining six criteria assigned one point each. A patient with a score of 1–6 is least likely to require surgery while a patient with a score of 7–10 is more likely to require surgery.^3^ The AAST grade includes whether the appendix is inflamed, gangrenous, or perforated. The grade ranges from I to V, with grade I the least severe and grade V the most severe.^4^ Patients diagnosed with AA were grouped according to time period, between March 1, 2018–February 29, 2020 as the pre-pandemic group and between March 1, 2020–February 28, 2021 as the pandemic group.

**Figure 1.**
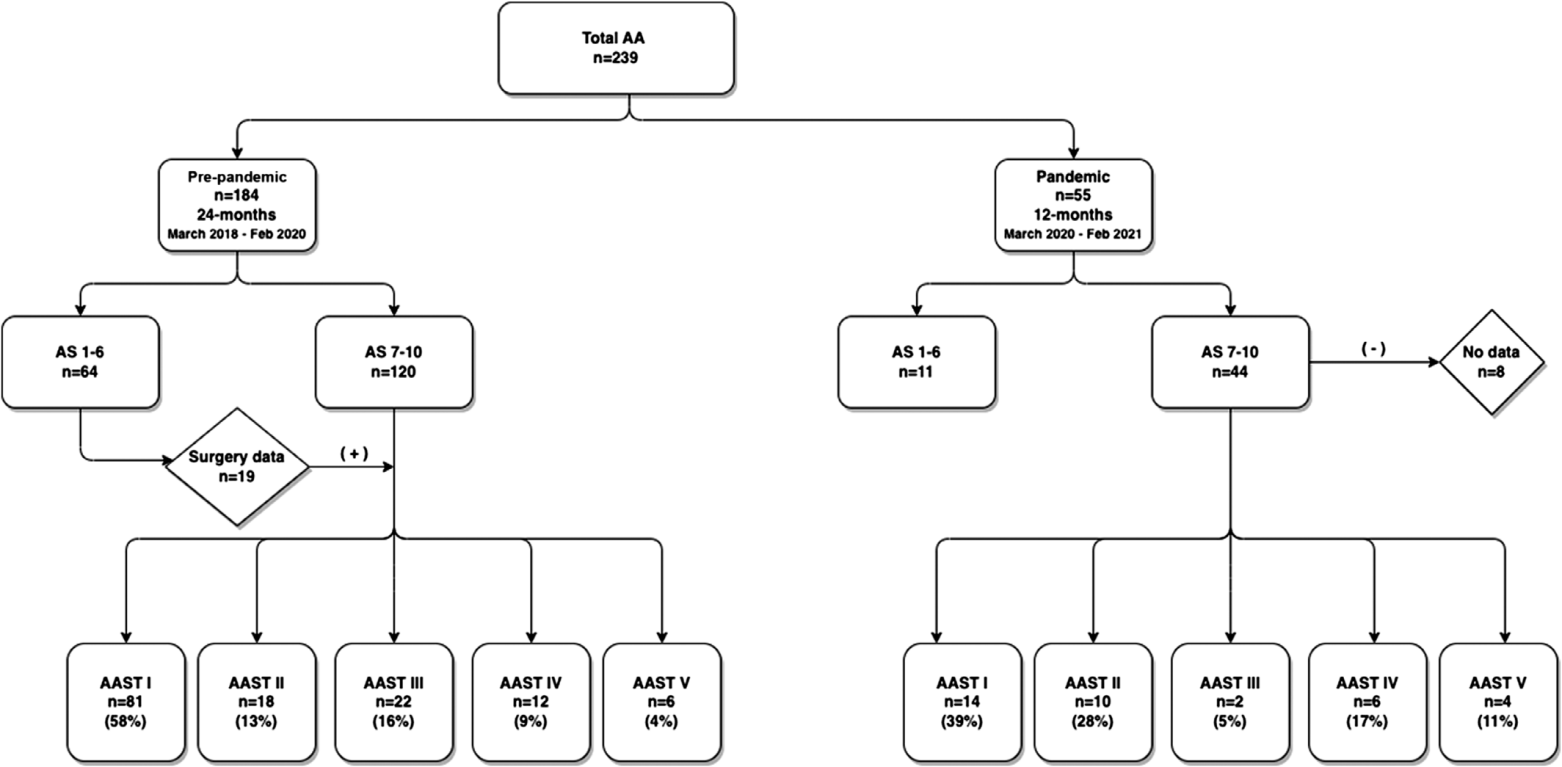
Flowchart of patients reviewed before and during COVID-19 pandemic. *AA*, acute appendicitis; *AS*, Alvarado score; *AAST*, American Association for the Surgery of Trauma grade.

We included patients aged 2–18 years who were diagnosed with AA between March 1, 2018–February 28, 2021 (Figure 1). Data were collected in adherence to the methodological standards proposed for medical record review studies by Worster et al.^5^ We used AS to determine incidence^3^ and AAST to determine severity.^4^ We analyzed the data using SPSS version 22 software (SPSS Inc, Chicago, IL). Independent sample *t*-tests, chi-square tests and Fisher exact tests were used for between-group analyses; *P* < 0.05 was considered significant.

## RESULTS

### Demographics

We reviewed 239 patient charts. There were 184 (77%) patients in the pre-pandemic group and 55 (23%) in the pandemic group. The average number of patients per year in the two years preceding the pandemic was 92 (38% per year) compared to 55 (23%) in the first pandemic year, a decline of 40%. The mean age of patients in our study group was 10.2 ± 3.9 years. Male gender was predominant (60%). The majority of our patients were of Hispanic or Asian ethnicity, reflective of our Queens community. Mean age, gender, and ethnicity were similar for pre-pandemic and pandemic groups.

### Alvarado Score

In both the pre-pandemic and pandemic groups, tenderness in the right lower quadrant was the primary clinical finding, and leukocytosis with left shift was the most frequent laboratory finding. An AS of 7–10 was more likely in patients in the pandemic group. Pre-pandemic AS compared to pandemic AS was significant (OR 2.13, 95% CI 1.03–4.41; *P* = 0.04).

### Ultrasound of the Appendix

We obtained an imaging study in 53% of patients in the pre-pandemic group and in 47% of patients in the pandemic group, *P* = 0.39.

### American Association for the Surgery of Trauma Grade

The AAST was applied to those with AS 7–10 and to an additional 19 patients with AS <7 and surgical data (total of 139 in the pre-pandemic group, 36 in the pandemic group). In the pandemic group, eight patients (15%) were transferred to a tertiary care center. The majority of patients in the pre-pandemic group had grade I, and the least number of patients were categorized with grade V. Most of the patients in the pandemic group had grade I and grade II severity and the least had grade III and grade IV severity. Chi-square analysis revealed a significant difference in the AAST severity grade distributions between pre-pandemic and pandemic groups (OR 0.36, 95% CI 0.1–1.35, *P* = 0.02) (Table 1). Only three patients tested positive for SARS-CoV-2.

**Table 1.** Alvarado score and American Association for the Surgery of Trauma grade before and during the COVID-19 pandemic.

Variables	Pre-pandemic n = 184 (24-months) n (%)	Pandemic n = 55 (12-months) n (%)	*P*-value
Demographics			
Age (years) mean (SD)	10.21 (3.9)	10.2 (3.9)	0.85
Gender			0.84
Male	111 (60.3)	34 (61.8)	
Female	73 (39.7)	21 (38.2)	
Ethnicity			
Caucasian	6 (3.3)	1 (1.8)	0.58
Hispanic	134 (72.8)	45 (81.8)	0.18
African American	6 (3.3)	3 (5.5)	0.45
Asian	36 (19.6)	6 (10.9)	0.14
Other	2 (1.1)	0 (0)	0.67
Alvarado score					
Migration of pain	84 (48.3)	34 (64.2)	0.04^*^
Anorexia	72 (52.9)	32 (69.6)	0.05^*^
Nausea	142 (78.9)	42 (80.8)	0.77
Tenderness in right lower quadrant	179 (97.8)	55 (100)	0.27
Rebound pain	44 (27.7)	17 (38.6)	0.16
Left shift	178 (96.7)	54 (98.2)	0.58
Fever	77 (41.8)	29 (52.7)	0.15
Leukocytosis	159 (86.4)	51 (92.7)	0.41
Total Alvarado score			0.04^*^
1–6	64 (34.8)	11 (20.0)	
7–10	120 (65.2)	44 (80.0)	
American Association for the Surgery of Trauma (AAST) grade
I: Acute inflamed appendix intact	81 (58.3)	14 (38.9)	0.37
II: Gangrenous appendicitis intact	18 (12.9)	10 (27.8)	0.31
III: Perforated appendix with local contamination	22 (15.8)	2 (5.6)	1.00
IV: Perforated appendix with phlegmon or abscess	12 (8.6)	6 (16.7)	1.00
V: Perforated appendix with generalized peritonitis	6 (4.3)	4 (11.1)	1.00
Total AAST grade			0.02^*^

* P < 0.05 was significant.

## DISCUSSION

Acute appendicitis is the most common abdominal surgical emergency in pediatrics and generally occurs frequently in males 10–19 years in age.^6^^,^^7^ The typical presentation includes periumbilical pain that migrates to the right lower quadrant of the abdomen associated with fever between 37.2–38.0° Celsius, nausea, loss of appetite, and diarrhea. Since these symptoms mimic other conditions, diagnosis of AA can be challenging. The AS assists in the diagnosis of AA, and visualization of the appendix on ultrasound is confirmatory. Delay in diagnosis can result in rupture of the appendix.^6^ Early surgical intervention is associated with lower risk of perforation. However, there are ongoing studies investigating whether appendectomy can be delayed by 12-24 hours and the role of non-operative management with the use of antibiotics as an effective treatment alternative.^7^ An AS of ≥7 is more likely to require surgery.^3^ The AAST anatomic grading system for appendicitis is a validated tool to assess severity. A higher AAST grade is associated with most severe cases and higher complication rates.^4^ We used the AS to determine incidence and the AAST grade to determine severity. Each patient was assigned to the highest group for analysis.

The WHO declared the COVID-19 pandemic in March 2020 as the virus spread worldwide.^8^ Measures to mitigate the spread of the virus modified the behavior of patients and the delivery of healthcare.^9^ The fear of exposure resulted in decline in pediatric routine and ED visits.^1^^,^^2^^,^^10^ Emergency departments were overwhelmed with COVID-19 cases, while the remainder of the hospital and medical staff was reorganized to care for those who were admitted. Elective surgeries were canceled, and non-urgent surgeries were delayed.^11^ Studies have reported the possible effect of the COVID-19 pandemic on presentation, management, and outcomes on appendicitis.^12^^–^^16^ A small percentage (5%) of our patients with AA tested positive for SARS-CoV-2, despite a reported possibility of AA as a post-inflammatory complication of SARS-CoV-2 infection.^17^^,^^18^

In our study, the median age and gender of our patients were concordant with known epidemiology of AA. The number of patients in each ethnic group is reflective of our community. The mean number of patients with AA per year pre-pandemic compared to pandemic declined by 40%, similar to findings by the US Centers for Diseases Control and Prevention.^19^ For each clinical criterion on the AS, pre-pandemic and pandemic groups were not significantly different. The percentage of ultrasound of the appendix performed was lower in the pandemic group and was also not significantly different. However, the higher number of patients with AS ≥7 in the pandemic group was significant. All our patients with AS 1–6 were managed with antibiotics. In both groups, only 66% of patients had complete surgical and pathological data. The percentage of grades I and II was higher in the pre-pandemic group, and the percentage of grades IV and V was higher in the pandemic group. The higher AAST grade was significant in the pandemic group.

Other studies regarding incidence and severity of AA during the COVID-19 pandemic were variable. In one study, a lower incidence and a higher complication rate in adults with AA were observed in a multicenter study in the Netherlands during the same period studied by us.^20^ Lucero et al reported a reduction in ED visits in both children and adults across our country.^2^ We concur with a decline in ED visits for AA. Studies related to severity of AA in children were all retrospective and based on patients with delayed presentation and increased incidence of complications.^13^^,^^15^^,^^21^^,^^22^ We did not correlate delayed presentation with severity. Incidence of complications was assessed using ASST grade. Gerall et al reported in a small sample of 48 patients in the pandemic group with delayed presentation and increased severity based on clinical presentation and radiological imaging of perforation and intra-abdominal abscess.^23^ We did see an increase in perforation with abscess (grade IV) in our pandemic group compared to the study from Italy by La Pergola et al in which the pandemic group had overall decreased hospital admission and unchanged number of complicated appendectomies as an indication of severity,^24^ and to the study from Lithuania by Vansevičienė et al when a surgical delay of four hours was not associated with increased complications.^25^ Not all studies confirmed delay in presentation or demonstrated increased severity of AA during the pandemic.^13^^,^^26^ There may be unmeasurable factors contributing to the changes seen during the peak of the pandemic affecting the behavior and relationship of our patients and healthcare systems.

## LIMITATIONS

This was a retrospective study conducted in two community, non-profit, teaching hospitals. We focused on the first year of the pandemic in NYC when the COVID-19 vaccine was limited for the age group studied and after worldwide spread. Change in medical coverage and parental health literacy were not taken into consideration when evaluating patient behavior. We did not include data on time from ED to operating room, outcomes, or follow-up after hospital discharge.

## CONCLUSION

In our sample from two urban community hospitals, there was a decline in the number of AA visits in our pediatric EDs. The severity of acute appendicitis in children and adolescents who presented to the ED was heightened during the COVID-19 pandemic. Clinicians need to be aware of increased severity when evaluating AA and possible increased risk of complications during a public health emergency. The increasing use of telemedicine to screen patients and of social media to obtain health information by patients fueled by the COVID-19 pandemic may contribute to the changing presentation of many conditions, including AA.

## Supplementary Information


