# Supplementary material for: Comparison of Pediatric Acute Appendicitis Before and During the COVID-19 Pandemic in New York City
Source: West J Emerg Med. 2023 Aug 22;24(5):956–61. doi: 10.5811/westjem.59393 (PMC10527842; doi:10.5811/westjem.59393)
Supplement: Supplementary file 1 [file wjem-24-956-s001.docx]

# Appendix

# Alvarado Score (adapted from Ohle et al).

| Feature | Score |
| --- | --- |
| Migration of pain | 1 |
| Anorexia | 1 |
| Nausea | 1 |
| Tenderness in right lower quadrant | 2 |
| Rebound pain | 1 |
| Elevated temperature | 1 |
| Leukocytosis | 2 |
| Shift of white blood cell count to left | 1 |
| Total | 10 |

**American Association for the Surgery of Trauma grade** (adapted from Finnesgard et al).

| Grade | Operative AAST description of appendicitis |
| --- | --- |
| Normal | Normal appendix |
| Grade I | Acutely inflamed, appendix intact |
| II | Gangrene, appendix intact |
| III | Perforated appendix with local contamination |
| IV | Perforated appendix with periappendiceal phlegmon or abscess |
| V | Perforated appendix with generalized peritonitis |

# *AAST*, American Association for Surgery of Trauma.


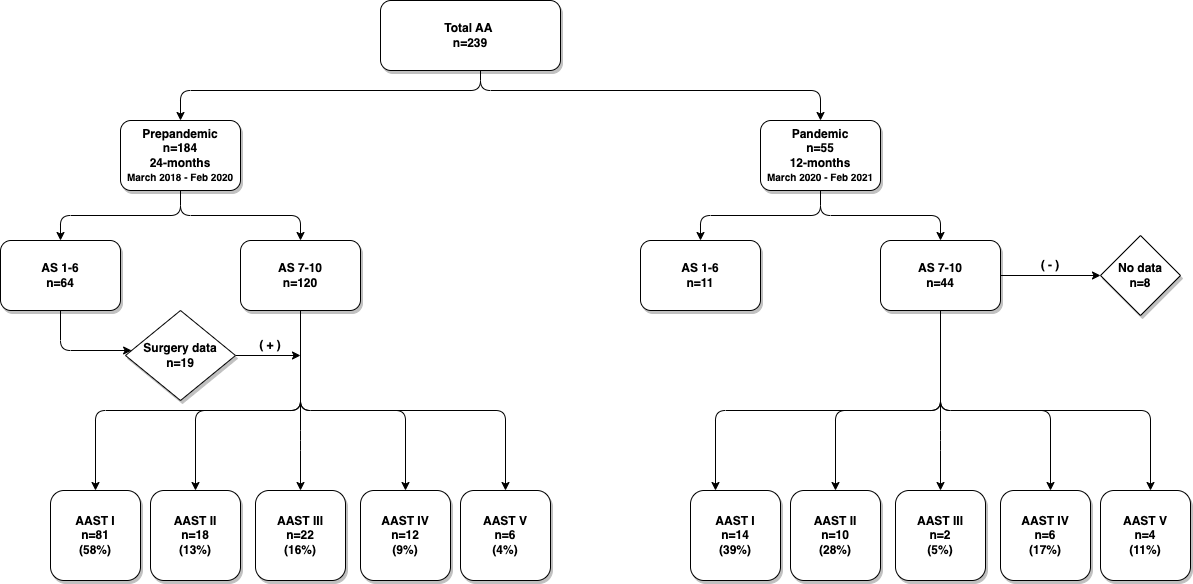


**Figure 1.** Flowchart of patients reviewed before and during COVID-19 pandemic.

*AA*, acute appendicitis; *AS*, Alvarado score, *AAST*, American Association for the Surgery of Trauma grade.

**Table 1.** Alvarado score and American Association for the Surgery of Trauma grade before and during the COVID-19 pandemic.

| **Variables** | | | **Pre-pandemic**  **n = 184**  **(24-months)**  **n (%)** | **Pandemic**  **n = 55**  **(12-months)**  **n (%)** | | ***P*-value** |
| --- | --- | --- | --- | --- | --- | --- |
| **DEMOGRAPHICS** | | |  |  |  | |
| Age (years) mean (SD) | | | 10.21 (3.9) | 10.2 (3.9) | 0.85 | |
| Gender | | |  |  | 0.84 | |
| Male | | | 111 (60.3) | 34 (61.8) |  | |
| Female | | | 73 (39.7) | 21 (38.2) |  | |
| Ethnicity | | |  |  |  | |
| Caucasian | | | 6 (3.3) | 1 (1.8) | 0.58 | |
| Hispanic | | | 134 (72.8) | 45 (81.8) | 0.18 | |
| African American | | | 6 (3.3) | 3 (5.5) | 0.45 | |
| Asian | | | 36 (19.6) | 6 (10.9) | 0.14 | |
| Other | | | 2 (1.1) | 0 (0) | 0.67 | |
|  | | |  |  |  | |
| **ALVARADO SCORE** | | |  |  |  | |
| Migration of pain | | | 84 (48.3) | 34 (64.2) | 0.04* | |
| Anorexia | | | 72 (52.9) | 32 (69.6) | 0.05* | |
| Nausea | | | 142 (78.9) | 42 (80.8) | 0.77 | |
| Tenderness in right lower quadrant | | | 179 (97.8) | 55 (100) | 0.27 | |
| Rebound pain | | | 44 (27.7) | 17 (38.6) | 0.16 | |
| Left shift | | | 178 (96.7) | 54 (98.2) | 0.58 | |
| Fever | | | 77 (41.8) | 29 (52.7) | 0.15 | |
| Leukocytosis | | | 159 (86.4) | 51 (92.7) | 0.41 | |
| Total Alvarado score | | |  |  | 0.04* | |
| 1-6 | | | 64 (34.8) | 11 (20) |  | |
| 7-10 | | | 120 (65.2) | 44 (80) |  | |
|  | | |  |  |  | |
| **AMERICAN ASSOCIATION for the SURGERY of TRAUMA (AAST) Grade** |  | | |  |  | |
| I: Acute inflamed appendix intact | | 81 (58.3) | | 14 (38.9) | 0.37 | |
| II: Gangrenous appendicitis intact | | 18 (12.9) | | 10 (27.8) | 0.31 | |
| III: Perforated appendix with local contamination | | 22 (15.8) | | 2 (5.6) | 1.00 | |
| IV: Perforated appendix with phlegmon or abscess | | 12 (8.6) | | 6 (16.7) | 1.00 | |
| V: Perforated appendix with generalized peritonitis | | 6 (4.3) | | 4 (11.1) | 1.00 | |
| Total AAST Grade | |  | |  | 0.02* | |
|  | |  | |  |  | |

# *P <0.05 was significant.
